# Supplementary material for: The Global Deterioration Scale for Down Syndrome Population (GDS-DS): A Rating Scale to Assess the Progression of Alzheimer’s Disease
Source: Int J Environ Res Public Health. 2023 Mar 14;20(6):5096. doi: 10.3390/ijerph20065096 (PMC10049652; doi:10.3390/ijerph20065096)
Supplement: Supplementary file 1 [file ijerph-20-05096-s001.zip › ijerph-2161383-supplementary.pdf]

Supplementary Materials

Comparisons of the probability and effect sizes effect values by consecutives groups of the GDS-DS stages.

Table S1. Effect sizes between groups of the GDS-DS stages.

	**GDS-DS stages**											
	**1-2**			**2-3**			**3-4**			**4-5**		
	**U**	**P**	**PS_{est}**	**U**	**P**	**PS_{est}**	**U**	**P**	**PS_{est}**	**U**	**P**	**PS_{est}**
BRIEF- P												
BRI index	64,500	0.395	**0.60**[a]	149.00	0.061	**0.67**[b]	87.500	0.183	0.61	57.500	0.567	0.51
CAMCOG-DS												
Total score	79.500	0.938	0.51	91.500	**0.010***	**0.80** [c]	70.500	**0.027***	**0.69** [b]	38.000	0.088	**0.68** [b]
Abstract thinking	71.00	0.595	0.56	166.00	0.139	**0.63** [a]	110.000	0.549	0.51	41.000	0.129	0.65
TB-DI												
Orientation	71.00	0.314	0.62	97.000	**0.002****	**0.78** [c]	13.000	**0.000*****	**0.94**[c]	33.500	0.057	**0.71** [c]
Free delay memory (stories)	61.500	0.775	0.53	207.000	0.663	0.54	101.000	0.331	0.55	52.000	0.467	**0.56** [a]
Semantic fluency (eat/drink)	75.500	0.856	0.52	180.000	0.265	0.60	114.000	0.906	0.49	43.500	0.114	**0.63** [a]
Formal fluency	77.500	0.364	0.61	123.000	**0.011***	**0.73** [c]	76.500	0.118	**0.66** [b]	66.000	0.716	0.44
Visual discrimination	63.500	0.733	0.54	189.000	0.367	0.58	99.000	0.363	**0.56** [a]	49.000	0.305	0.58

GDS-DS, global deterioration scale for people with Down's syndrome ; BRIEF-P, behavior rating inventory of executive function parents form; BRI, behavioral regulation index; CAMCOG-DS, Cambridge cognitive examination for older adults with Down's syndrome and other intellectual disabilities; BT-DI, Barcelona test for intellectual disability; U, Mann–Whitney U test; P, *p* value; PS_{est}, probability of superiority effect size; * $p < 0.05$, ** $p < 0.01$, *** < 0.001; [a] $PS_{est} \geq 56$, [b]$PS_{est} \geq 0{,}64$, [c]$PS_{est} \geq 0{,}71$. Significant effect sizes and *P* values with progressive decrease on the performance across the stages (Table 7) in bold.
